# Psychosocial experiences of pregnant women living with HIV in Ibadan, Oyo State

**DOI:** 10.21203/rs.3.rs-3327673/v1

**Published:** 2023-09-12

**Authors:** Folahanmi Akinsolu, Abisola Lawale, Samuel Bankole, Zaniab Adegbite, Ifeoluwa Adewole, Olunike Abodunrin, Mobolaji Olagunju, Oluwabukola Ola, Abel Chukwuemeka, Aisha Gambari, Abideen Salako, Oliver Ezechi

**Affiliations:** Lead City University; Lead City University; Lead City University; Lead City University; Lead City University; Lagos State Health Service Commission; Nanjing University; Lead City University; Lead City University; Nigerian Institute of Medical Research; Nigerian Institute of Medical Research; Nigerian Institute of Medical Research

**Keywords:** Women, Psychological Vulnerabilities, Antiretroviral Therapy, HIV Care, Mental Health Evaluations

## Abstract

**Introduction:**

Pregnancy often intensifies psychological vulnerabilities in women living with HIV (WLHIV) due to increased stressors such as health concerns, infant infection risks, and the management of special neonatal needs like prophylactic antiretroviral care.

**Methodology:**

The study was conducted in four HIV treatment centres with participant selection based on the following criteria: an Edinburgh Postnatal Depression Scale (EPDS) score of 13 or above, gestational age between 14 to 40 weeks, less than five years of antiretroviral therapy (ART) usage, and history of partner conflict. This research forms a more extensive study of stress and depression amongst pregnant and postpartum WLWH. In-depth interviews, ranging from 20 to 40 minutes, were conducted with 26 HIV-positive pregnant women in private rooms within selected antiretroviral clinics from October and December 2022.

**Results and Discussion:**

The study discovered that the support received from healthcare providers concerning ART and Prevention of Mother-To-Child Transmission (PMTCT) practices alleviated women’s fear of death and perinatal transmission which bolstered their involvement in HIV care and fostered the birth of children not infected with HIV. Women perceived monogamy as a protective measure against HIV contraction. Participants who reported having partners engaging in unsafe sexual practices expressed anger and blame. The observation of other women with similar experiences aided in coping mechanisms, reaffirming previous findings that knowing someone living with HIV helps to accept their status due to the comfort derived from shared experiences.

**Conclusion:**

Healthcare providers attending to pregnant WLWH can alleviate psychological distress by reinforcing positive coping strategies. These include consistent psychological distress screenings in HIV clinics and relevant mental health evaluations with appropriate care referrals.

## Introduction

Globally, the human immunodeficiency virus (HIV) is regarded as a public health issue of great concern and disproportionately burdens developing countries. Sub-Saharan Africa (SSA) accounts for 71% of the global population of people living with HIV (PLWH) [1]. In SSA, more women live with HIV (WLWH) than men, as women account for 58% of the PLWH [2]. In 2020, Nigeria was estimated to have 1.9 million PLWH making Nigeria the country with the third highest burden of HIV.

The prevalence rate of HIV was also the highest among the female adult population, at 1.6% [3]. Contributing factors to the higher HIV burden among women are both physiological [4] and socioeconomic [5]. Women face gender inequality which makes them vulnerable to gender-based violence and limits their options for negotiating safe sex in intimate relationships [6–8].

Although the developments in ART treatment have significantly reduced living with HIV to a causal relationship between disease and treatment, this circumstance makes WLWH more susceptible to various stressors that can occur over time. These stressors potentially harm a person’s psychological well-being and quality of life [9,10]. Mental health-related variables could compromise ART’s efficacy and negatively impact women’s quality of life and mental health [11,12].

Women are most often made aware of their positive HIV diagnosis during pregnancy. When first diagnosed with HIV, they experience intense emotions that can disrupt their lives [13]. For WLWH, pregnancy can be a time of increased psychological vulnerability [14]. In addition to the usual strains of new motherhood, WLWH also has to deal with stressors related to their health, the potential infectious statuses of their infants, and taking care of their new-born’s particular needs, like providing prophylactic antiretroviral medication [15–17].

Particularly in Nigeria, where the availability of HIV and mental health services are limited, it is essential to develop an understanding of the forms of psychosocial challenges faced by pregnant and postpartum WLWH and also how they cope with them to develop interventions to help reduce psychological distress and also support the progression of healthy pregnancies as well as postpartum outcomes.

Thus, this study aimed to explore the psychosocial experiences and challenges of prenatal women living with HIV in Ibadan, Nigeria, and the measures through which they cope with the challenges.

## Materials and methods

### Study design

This study was conducted within a facility-based mixed-methods study investigating psychosocial outcomes and experiences of pregnant women living with HIV in Ibadan, Nigeria. The study involved the collection of both quantitative and qualitative data to develop an enhanced understanding of the psychosocial health of women living with HIV during pregnancy and postpartum, the intricacies of their experiences as well as their coping strategies. Women living with HIV during pregnancy and postpartum were recruited from four selected sites offering ART services between October and December 2022. The quantitative data for the study was collected through interviewer-administered questionnaires, and a sub-sample of women living with HIV took part in in-depth qualitative interviews (n = 26).

### Study setting

The study was conducted in four (4) HIV treatment centers in Ibadan, which were purposively selected. The centres are; State Hospital, Adeoyo, Ringroad; Adeoyo Maternity Health Centre; St. Mary Catholic Hospital, Eleta; and St Annes Anglican Hospital, Molete.

### Participants

All the participants enrolled in the facility-based study were recruited to participate in the survey and interviews within the same period from the selected study sites. The women recruited to participate in the study were women living with HIV over the age of 18 that were currently pregnant and were attending any of the four selected antiretroviral treatment clinics in Ibadan, Oyo state, Nigeria. The study excluded women with existing mental illnesses or could not provide explicit consent.

Purposive sampling was used to select participants based on their responses to the Edinburgh Postnatal Depression Scale (EPDS) that was used to assess for symptoms of depression (EPDS ≥ 13), gestational age of 14–40 weeks, less than five on ART and history of conflict with a partner, within the parent study that focused on prevalence and correlates of depression and perceived stress amongst women living with HIV during the pregnancy and postpartum period.

A total of thirty-eight pregnant women were identified to have a score of 13 or more in the EPDS (A score of 13 or more is interpreted as “suffering from a depressive illness of varying severity”) and also had both gestational age of 14–40 weeks, less than five years on ART and history of conflict with a partner which showed significant association with perinatal depression according to the parent study. The in-depth interviews were conducted with a total of twenty-six respondents that were willing to be interviewed after.

### Data collection

In the parent study, participants were provided with information sheets outlining the objective and scope of the study. The contents of the information sheets were also read and explained to the participants, after which the participants were allowed to ask questions. Upon acceptance to participate in the research, they were given consent forms to sign. Participants were informed of their right to withdraw from participating in the study at any time they wished to do so. They were also given assurance of their confidentiality.

The in-depth interviews were conducted among HIV-infected pregnant women, allowing the different issues related to this research area to be covered. Twenty-six interviews were conducted between October and December 2022with HIV-infected pregnant women attending any of the four antiretroviral treatment clinics. All interviews were conducted face-to-face with the participants in private rooms in the selected treatment clinics. Each interview lasted for about twenty to thirty minutes. Interview participants were reminded of the content of the information sheet given to them during the quantitative phase of the study and of their right to withdraw from participating. Their approval was also requested to record the interview. The interview guide included inquiries concerning WLWH’s pregnancies and postpartum experiences, women’s attitudes toward getting pregnant, how their HIV status affected those attitudes, and their partners’ perceptions of the pregnancy. Probes were used to clarify and help elaborate questions throughout the interviews, thus expanding the women’s stories within the larger narrative.

### Data analysis

The interview audio recordings were translated and transcribed verbatim into English. The transcripts were then examined to evaluate the accuracy and integrity of the translation. NVivo 12 (QRS International) was used to facilitate analyses. Initial data analysis was done using content analysis to investigate the experiences of depression among WLHIV during pregnancy and postpartum and their coping strategies. Transcripts were read to identify significant themes and to inform the development of a coding scheme to categorize the data. The final coding scheme included both a priori themes and those which emerged from preliminary readings of the transcripts. A codebook was created to guide the coding process. Data reduction approaches extracted the overarching narrative from the most relevant facts.

### Ethics Consideration

This study was conducted following the ethical principles of the Declaration of Helsinki. Ethics approval was obtained from the Oyo State Ministry of Health ethics committees (AD 13/479/44539) and the Lead City Health Research and Ethics Committee (LCU-REC/22/125). Before administering the questionnaire, the participants were given information sheets outlining the study’s objective and scope, which were duly explained to the participants in English or the local dialect (Yoruba/Pidgin). The participants were informed that participation in the study was voluntary and that they were free to withdraw from the study at any point without any consequences. The confidentiality and anonymity of the participants were ensured, and all data were kept confidential and were used only for research purposes. The participants were assured that participation or non-participation would not affect their access to healthcare services. In addition, participants who required psychological support after the study were referred to the appropriate healthcare professionals. The interview audio files were transferred onto a password-protected USB stick and computer and deleted from the recording device. After transcription, the audio files were deleted.

## Results

The total number of participants comprised 26 HIV-infected pregnant women, of whom 14 were pregnant, and 12 were in the postpartum phases. Participants aged 22 to 43 years received lifelong ART. The majority of the participants (n = 16) were married, and all of the participants were employed. All of the participants were observed to be Christians in terms of religion. (See Table 2)

### Themes

Following the thematic analysis of the interviews, four significant themes emerged as major issues experienced by WLWH during pregnancy and postpartum. The main themes included were Unexpected Diagnosis; Disclosure Issues; Emotional Distress; and Isolation. Also, three themes emerged as ways the women cope with adversity in the face of challenges. The main themes included Acceptance of Self and HIV Diagnosis; Social Support from Partners, Family, and Friends, and Support from the Healthcare Delivery System. (See Table 3)

#### Challenges

##### Theme 1.1. Unexpected Diagnosis

Most participants were shocked when they learned about their positive HIV diagnosis. Before the HIV diagnosis, the women did not believe that they could contract the HIV infection.

###### Theme 1.1.1. Fear because of Preconceptions of HIV

Some of the participants had misconceptions about the disease, and they perceived HIV as a disease that was synonymous with death which informed their feelings of fear, sadness, and grief upon diagnosis. A response supporting this was given by Participant 9, who said:

“I had heard the disease kills people and other things like that. That’s why I was crying. That’s why I was thinking so much that time.” **P9**

Learning that they had HIV was comparable to receiving a terminal illness diagnosis, generating uncertainty and hopelessness. Participant 12 shared that a leading factor in the generation of these feelings is the way being diagnosed with HIV is described by others:

“They normally use one harsh term in Yoruba, which demoralizes people with HIV. They say “won tilugbakokoroti o gbogun” (They have been struck with a disease that has no cure).” **P12**

###### Theme 1.1.2. Shock, Disbelief, and Denial of Diagnosis

The participants reported experiencing intense feelings of shock and sadness following the revelation that they had been diagnosed with the disease. This testament is similar to Participants 1 and 12 revelations.

“I don’t think that there’s a single person who would receive that kind of news so suddenly that wouldn’t feel as shocked as I was at that moment. It hit me so suddenly.” **P1**.

“I was devastated. I had almost gone mad. Almost, it was just a tiny line for me, if not for God’s grace. I thank God” **P12**.

With further exploration of the cause of this shock, a common finding was that the positive HIV test result was unexpected. The reasons range from having a previous negative result, having only one partner, and believing that positive results were associated with practicing unsafe sexual behaviors.

“When we got married, we did all the tests. And there was nothing like HIV. So, I didn’t understand how I came about it.” **P11**

“I did not have it in mind at all because I’m not a promiscuous person. I didn’t think I had done anything I wasn’t supposed to do somewhere, and I would have gotten it from there. I didn’t have it in mind at all that I could have the disease until they told me that I did.” **P7**

###### Theme 1.1.3. Anger towards the partner.

While the participants were upset with themselves for contracting the illness, some blamed it on their spouses. Participant 20 reported being aware of her infection and was angry and blamed her husband.

“I was very angry with him because I knew it was from him. He doesn’t know how to stay in one place. I even tried to fight and shout at him; it was like he was trying to kill me. I got even angrier when I thought our children could have it too.” **P20**

Participant 23 also expressed pain and unhappiness at being negatively affected by the sexual behavior of her partner.

“He does not know how to protect himself and does not like using condoms. Unfortunately, I had to be on the receiving end of that. It was very painful to think back on it.” **P23**

##### Theme 1.2. Disclosure of HIV Diagnosis

For some participants, their diagnosis was perceived as a personal burden they wished to deal with on their own. This perception was illustrated by Participant 12, who highlighted that it was essential to keep the HIV diagnosis a secret.

“I haven’t told anyone because it is our personal family issue. Nobody else needs to know about it.” **P12**

For the participants of this study, disclosure was selective. They only disclosed their HIV diagnosis and that of their child to persons they could rely on to keep the information a secret. Additionally, they disclosed to those they thought would support them.

“I was ashamed to tell her at first. But my mother and I were close, and I needed someone to talk to. And normally, there was nothing I couldn’t talk to her about. She was the only one I could turn to.” **P22**.

###### Theme 1.2.1. Fear of Disclosure.

Nearly all the participants expressed fear of disclosure, anxiety about HIV-related stigma, and rejection as critical concerns. Some women worried that disclosing their positive HIV status to their partners would lead to assumptions that they had been unfaithful to the partnership, and they feared the emotional and physical violence and isolation that may have followed. These fears were illustrated by Participant 4 in her report about disclosing her status to her partner:

“No, I did not tell him. I didn’t want more problems. If he knows about it, he can throw us out. Where would I go? And I cannot raise a child myself.” **P4**.

In addition to this, she also believed that others would not keep the information confidential and they would be rejected or treated differently:

“Before I tested positive, I had heard things people said about those with the disease. They say it’s incurable, and people that get it are dirty. I didn’t want anyone to look at me or my child like that. We would both be isolated. Nobody would want to come near us” **P4**.

###### Theme 1.2.2. The outcome of disclosure.

Some participants revealed that they experienced rejection and embarrassment resulting from disclosure to their families and sexual partners. Participant 13 narrated that she was humiliated, isolated, and ultimately rendered homeless by her partner and his family when she disclosed her positive diagnosis.

“He told everybody in his family that I had HIV. I felt so ashamed, and He told them I was sleeping around and wanted to give him a child that didn’t belong to him. I don’t stay with him anymore. They said I should leave.” **P13**.

In contrast, some participants reported receiving support from those close to them after disclosure, which was evident from Participant 16, who said:

“There’s so much stigmatization and false information regarding HIV, but they are very supportive. Even my daughter checks with me to make sure I’m fine.” **P16**.

Participant 6 reported that her partner was not supportive upon initial disclosure but became more accommodating and supportive after the counseling intervention from the health facility workers she was attending.

“He reacted the way you would expect anybody to react when you hear that; what kind of thing is this? But I’ll never forget that he focused more on my and our child’s health. His love for me made him take things more calmly.” **P6**.

##### Theme 1.3. Emotional Distress

Participants described the impact of being diagnosed with HIV as psychological and emotional. The majority of the participants reported that being pregnant added to their anxiety. They worried their unborn child might contract HIV, which caused them pain.

###### Theme 1.3.1. Fear for the life of their child.

Participants, upon the discovery of their HIV diagnosis during their pregnancy, were concerned about the prospect that their unborn child would contract HIV and feared that their babies would die soon after birth due to HIV.

“I was about to have a baby, and I wanted to have more children after that. I didn’t understand it because I thought they were trying to tell me that I could not have children or my baby would die.” **P3**.

One of the participants who were aware of her diagnosis before her pregnancy shared the same fears before she got pregnant and as well as after:

“I thought about it a lot that time. Before and after I got pregnant, I was thinking, would I be able to have a child? And when I got pregnant, I was thinking, will this child live or die?” **P10**.

###### Theme 1.3.2. Guilt, Shame, and Self-blame.

Many participants expressed sentiments of shame and guilt. They reported being ashamed of their diagnosis and viewed themselves as pariahs.

“I don’t know what anybody else thinks, I can’t speak for them, but for me, it’s bad.” **P19**.

They also felt responsible for subjecting their child to the possibility of living with HIV and thus blamed themselves:

“I realized that these tattoos I drew on my body were the most likely cause. And I was thinking that if I didn’t do that to myself, I wouldn’t be here and sick like this because I couldn’t let anyone follow me so they wouldn’t find out. I still look at them regretfully because now I’m suffering for it, and my child might have to suffer too.” **P10**.

##### Theme 1.4. Isolation.

In this study, stigma and the fear of stigma were pervasive in these women’s lives and had a significant impact. Some of the participants experienced stigma from healthcare providers as well as family members upon disclosure. Being diagnosed with HIV also made these women wilfully withdraw and isolate themselves, resulting from the fear of rejection and of the prejudice of the people around them.

###### Theme 1.4.1. Stigmatization.

When seeking antenatal care services at the hospital, some of the study’s participants were subjected to stigma and discrimination and reported receiving less favorable treatment from medical professionals than HIV-negative women.

“Even though I work in a hospital environment, when people see you have it, they…, actually on your case note they would write it there on your case note. They would use that sign, the red positive; they would put it there to mark that thing. Everyone in that field would know that the person is HIV positive and treat you somehow.” **P12**.

Stigma was also reported from several sources, including family members.

“My sister thought I was going to die. That was the first thing she shouted about. It wasn’t until I tried to calm her down and explain to her. But even then, she has shifted away from me; she’s not as free with me as she used to be. I noticed that she doesn’t let her children come near me.” **P9**.

###### Theme 1.4.2. Self-isolation.

For most participants, their diagnosis contributed to feelings of low self-worth, and they felt the need to isolate themselves from their immediate environment. This self-isolation may also be perceived as means of coping with the fear of stigmatization.

“I was withdrawn, I didn’t go to parties, I didn’t mingle, I discontinued associating with all my friends.” **P19**.

“I don’t talk to people, even those who attend this clinic with me. I don’t have friends here.” **P7**.

#### Coping Strategies

##### Themes 2.1. Acceptance of Self and HIV Diagnosis.

Participants in this study experienced internal stigma connected to HIV, which caused them to withdraw from their community. Participant 12 explains stated that,

“I was withdrawn, I didn’t go to parties, I didn’t mingle, I discontinued associating with all my friends. It affected me even up till today because a majority of the friends I had then, I have lost their contacts; I don’t have any information about them.” **P12**.

However, viewing HIV as a disease affecting many people rather than a death sentence and accepting her HIV status was a decisive step towards self-acceptance. She later says:

“If someone having diabetes can be living on drugs, someone that is hypertensive can be living on drugs, someone with hepatitis can be living on drugs, then what is the big deal about living with HIV that you cannot just continue living in your drugs and you will be doing very fine. And life goes on.” **P12**.

##### Themes 2.2. Social Support from Partners, Family, and Friends.

Some participants indicated that having their partners’ and other family members’ support influenced their pregnancy experience and postpartum results beneficially. The participants described how their partner and family members were committed to helping them adhere to ART and PMTCT protocols:

“He is resigned about it now. He has accepted it. Initially, it wasn’t easy, but we thank God. Now there is no problem at all. He even reminds me to take my medication.” **P3**.

“She has helped me so much with my child. He’s even with her now in Ondo. I didn’t want my husband to question why my child was taking medication, so I was with my mother from giving birth until I stopped breastfeeding.” **P4**.

##### Themes 2.3. Support from Healthcare Delivery System

Some women could overcome their concerns about perinatal transmission, seek HIV care, and give birth to an HIV-uninfected child with the assistance of reliable healthcare professionals regarding PMTCT practices.

“They told me my baby could be healthy without the disease. That was all I wanted. They told me that I could have a healthy baby that wouldn’t even have HIV, and that was a relief for me.” **P1**.

###### Themes 2.3.1. The role of counseling.

The participants emphasized the importance of regular counseling. In their opinion, counseling was essential to understand their situation. They also admitted that the counseling they got had given them hope for a future.

“That was where they counseled me. They told me there was no problem, that I should calm down and nothing would happen. It helped me a lot. They explained that having this disease doesn’t mean I will die. They told me I could live a healthy life if I took my medication” **P2**.

Some participants also talked about how helpful interacting with other WLWHs within the healthcare facility was.

“And I had also met some people with the disease that had a baby, and nothing was wrong with the baby, so I didn’t feel anyhow. I wasn’t afraid.” **P8**.

###### Themes 2.3.2. Confidence in the use of ART and Adherence.

The participants’ faith in ART served as a coping mechanism after receiving their HIV diagnoses. The women believed that they and their children could have everyday lives because of the availability of effective antiretroviral medications.

“I’m taking my drugs religiously, and my viral load is so low, I cannot infect you. I would tell you there’s no big deal, I can have a child, you have a child, and I have a child, your child is normal, and my child is normal, you can breastfeed, and I can breastfeed.” **P12**.

“But they told us everything won’t affect the baby if we keep using the medication. And I made sure I took my medication regularly.” **P11**.

## Discussion

The diagnosis of HIV represented a traumatic turning point for the participants, triggering intense emotions that align with previous research [15,16]. Many women reacted with disbelief, refusing to accept their HIV-positive status [17,18]. This denial stemmed primarily from the women’s self-perception as low-risk individuals for HIV transmission. Their understanding of HIV was anchored mainly on stereotypes, linking the disease to promiscuous behavior and underestimating its prevalence in monogamous relationships [19,20]. Notably, the women aware of their partners’ risky behaviors expressed anger and blamed their partners for their diagnosis.

Disclosing their HIV status was daunting for many women in this study. They feared a spectrum of adverse reactions, including domestic violence, neglect, divorce, and psychological abuse, a finding that is consistent with existing literature [21]. The HIV diagnosis thus served as a source of distress, initiating a cycle of fear and worry about their health and that of their unborn children. The women also expressed concerns about their ability to care for their children due to their compromised health, aligning with concerns reported in previous research [22].

The anxiety of potentially transmitting the virus to their unborn child was particularly burdensome, a finding corroborated by previous research indicating elevated maternal anxiety levels before confirming the child’s HIV status [23]. The emotional burden was also prolonged, with the worry about the child’s health persisting throughout the HIV testing period for the infant [23,24], which underscored the urgency of providing timely psychosocial support to these women to alleviate fears of vertical transmission and promote engagement in HIV care [26].

In reaction to their diagnosis, several women reported self-imposed isolation or withdrawal from social activities, consistent with previous studies [26,27]. They described stigmatizing experiences, including open criticism from healthcare professionals, further alienating them from accessing care, which suggests a gap in the HIV and antenatal clinics’ approach to the psychosocial needs of HIV-positive pregnant and postpartum women [28]. Therefore, integrating psychosocial care within PMTCT programs is critical in facilitating acceptance of self and HIV diagnosis [29].

As the women navigated the emotional tumult of living with HIV, acceptance of their HIV status emerged as a central coping strategy. This acceptance signified a turning point in their journey, enabling them to reframe their situation and manage the subsequent challenges more effectively [30,31]. This acceptance process often occurred over time and was facilitated by several factors, including personal resilience, supportive relationships, and positive interactions with healthcare professionals.

The women’s narratives also revealed the crucial role played by their partners, families, and friends in helping them cope with their diagnosis. These individuals provided emotional support and practical assistance, ranging from companionship and encouragement to help with medication management and medical appointments [32,33,34]. Such social support networks have been documented in previous studies to buffer against psychological distress, promote treatment adherence, and improve overall health outcomes among people living with HIV [35,36].

However, while social support was beneficial, it was not universally available to all the women in the study. Some reported the absence of supportive relationships or reluctance to disclose their status due to fears of rejection or discrimination. These findings underscore the need for broader community-based interventions to reduce stigma, increase awareness, and foster supportive environments for women living with HIV.

In addition to their relationships, the women identified healthcare providers and systems as critical sources of support. The guidance they received regarding antiretroviral therapy (ART) and prevention of mother-to-child transmission (PMTCT) practices helped to alleviate fears related to their health and that of their babies. Providing accurate information and reassurance from health professionals was pivotal in their journey toward acceptance and mitigating fears about death and vertical transmission [37].

Beyond their interactions with healthcare providers, the participants also found solace and strength in connecting with other women living with HIV. This peer interaction, often facilitated within healthcare settings, provided much-needed reassurance, enabling them to see that it was possible to live with HIV and have healthy, HIV-negative children [38,39]. It also gave them a forum to share experiences, ask questions, and learn from others in similar situations.

Despite these supportive avenues, many women were reluctant to disclose their status to the broader community due to fears of stigma and discrimination, contrasting with previous findings suggesting that community support can be crucial in medication adherence and overall coping with the disease [28,29]. It underscores the complexity of these women’s social landscapes, marked by intersecting domains of stigma, disclosure, social support, and healthcare engagement.

In conclusion, these findings shed light on the multifaceted challenges and coping strategies among pregnant women living with HIV. They illustrate a complex trajectory marked by initial shock and denial, followed by a gradual acceptance process facilitated by personal resilience, social support, and effective engagement with healthcare services. However, they also highlight persistent barriers to social support and community engagement, underscoring the need for comprehensive interventions to reduce stigma and enhance supportive environments for these women. These insights can inform the development of more holistic, person-centered approaches to HIV care that address the physical health and emotional and psychosocial needs of pregnant women living with HIV.

## Figures and Tables

**Table 1:** Key interview themes and Sample Questions

Key interview themes	Sample Questions
Diagnosis	• How did you find out about your diagnosis? o How did you react to finding out?
Disclosure	• Whom did you tell first and why? o What was the reaction of this person? o Did you feel rejection or mistreatment? • Did anyone help you? Who did you go to when you needed support? • Did your diagnosis affect the way you interact with those around you?
Feelings towards pregnancy	• Did your diagnosis change your perception of your pregnancy? o If yes/no, why? • What challenges did you face during your pregnancy because of your HIV diagnosis?
Feelings towards the outcome of pregnancy	• Has your child been tested for HIV? • If yes, how did you feel about going for testing? • What was the result? How did it feel to learn the result?
Experiences following childbirth	• How would you describe your experience of living with HIV? • How did having this [pregnancy outcome] affect your life?

**Table 2:** Demographic data for interview participants

Demographic Information	Number of Participants (n = 26)
Age (Mean = 30.8 ± 5.6)	
18–24	7
25–34	12
>35	7
**Tribe**	
Yoruba	22
Igbo	4
Hausa	-
**Religion**	
Christianity	18
Islam	8
**Marital Status**	
Married	22
Separated	4
**Employment Status**	
Employed	23
Unemployed	3
**Educational Level**	
Primary	3
Secondary	16
Tertiary	7
**Perinatal Status**	
Postpartum	12

**Table 3.** Main themes and sub-themes for challenges and coping strategies

	Themes	Sub-themes
**Challenges**	Unexpected Diagnosis	Fear because of Preconceptions of HIV
		Shock, Disbelief, and Denial of Diagnosis
		Anger Towards Partner
	Disclosure Issues	Fear of Disclosure
		Outcome of Disclosure
	Emotional Distress and Fear	Fear for the life of their child
		Guilt, Shame, and SelfBlame
	Isolation	Stigmatization
		Self-isolation
**Coping Strategies**	Acceptance of Self and HIV Diagnosis	
	Social Support from Partners, Family, and Friends	
	Support from Healthcare Delivery System	The role of counselling
		Confidence in ART and Adherence

## Data Availability

The data used to support the findings of this study are available from the corresponding author upon request.

## References

[1] Waweru G, Korir J. The Impact of HIV/AIDS Expenditure on HIV/AIDS Incidence Rate in Sub-Saharan Africa.

[2] Hegdahl HK, Fylkesnes KM, Sandøy IF. Sex differences in HIV prevalence persist over time: evidence from 18 countries in sub-Saharan Africa. PloS one. 2016 Feb 3;11(2):e0148502. 26841112 10.1371/journal.pone.0148502 PMC4739589

[3] Luke A, Owhonda G, Ogbondah BO, Tobin-West CI. Associated factors and virologic outcomes of cisgender groups among people living with HIV/AIDS attending a tertiary health facility in Rivers State, Nigeria. International Journal of Community Medicine and Public Health. 2022 Oct;9(10):3948.

[4] Winkleby MA, Kraemer HC, Ahn DK, Varady AN. Ethnic and socioeconomic differences in cardiovascular disease risk factors: findings for women from the Third National Health and Nutrition Examination Survey, 1988–1994. Jama. 1998 Jul 22;280(4):356–62. 9686553 10.1001/jama.280.4.356

[5] Mitchell S, Shaw D. The worldwide epidemic of female obesity. Best practice & research Clinical obstetrics & gynaecology. 2015 Apr 1;29(3):289–99. 25487257 10.1016/j.bpobgyn.2014.10.002

[6] Mashiri L. Conceptualisation of gender based violence in Zimbabwe.

[7] Gordon SF, Collins A. “We face rape. We face all things”: Understandings of gender-based violence amongst female students at a South African university. African Safety Promotion: A Journal of Injury and Violence Prevention. 2013;11(2):93–106.

[8] Schulkind J, Mbonye M, Watts C, Seeley J. The social context of gender-based violence, alcohol use and HIV risk among women involved in high-risk sexual behaviour and their intimate partners in Kampala, Uganda. Culture, health & sexuality. 2016 Jul 2;18(7):770–84. 10.1080/13691058.2015.1124456 PMC4898159 26786739

[9] Schönnesson LN. Psychological and existential issues and quality of life in people living with HIV infection. AIDS care. 2002 Jun 1;14(3):399–404. 12042085 10.1080/09540120220123784

[10] Gore-Felton C, Koopman C. Behavioral mediation of the relationship between psychosocial factors and HIV disease progression. Psychosomatic Medicine. 2008 Jun 1;70(5):569–74. 18519885 10.1097/PSY.0b013e318177353e PMC11554426

[11] McAuley E, Morris KS. State of the art review: advances in physical activity and mental health: quality of life. American Journal of Lifestyle Medicine. 2007 Sep;1(5):389–96.

[12] Dalmida SG. Spirituality, mental health, physical health, and health-related quality of life among women with HIV/AIDS: integrating spirituality into mental health care. Issues in mental health nursing. 2006 Jan 1;27(2):185–98. 16418078 10.1080/01612840500436958 PMC3978566

[13] Kelly C, Alderdice F, Lohan M, Spence D. Creating continuity out of the disruption of a diagnosis of HIV during pregnancy. Journal of clinical nursing. 2012 Jun;21(11–12):1554–62. 22458718 10.1111/j.1365-2702.2011.04017.x

[14] Waldron EM, Burnett-Zeigler I, Wee V, Ng YW, Koenig LJ, Pederson AB, Tomaszewski E, Miller ES. Mental health in women living with HIV: the unique and unmet needs. Journal of the International Association of Providers of AIDS Care (JIAPAC). 2021 Jan 20;20:2325958220985665. 33472517 10.1177/2325958220985665 PMC7829520

[15] Contreras Carmen , “Emotional Experiences of Mothers Living With HIV and the Quest for Emotional Recovery: A Qualitative Study in Lima, Peru,” The Journal of the Association of Nurses in AIDS Care : JANAC 30, no. 4 (2019): 440–450. 10.1097/JNC.0000000000000051 PMC8324040 31241508

[16] Genevieve Marion Fords, Talitha Crowley, and Anita S Van der Merwe, “The Lived Experiences of Rural Women Diagnosed with the Human Immunodeficiency Virus in the Antenatal Period,” SAHARA-J: Journal of Social Aspects of HIV/AIDS 14, no. 1 (2017): 85–92. 10.1080/17290376.2017.1379430 PMC5639609 28949277

[17] enise Diaz Payán , “‘It Was as Though My Spirit Left, Like They Killed Me’: The Disruptive Impact of an HIV-Positive Diagnosis among Women in the Dominican Republic,” Journal of the International Association of Providers of AIDS Care (JIAPAC) 18 (January 1, 2019): 232595821984904. 10.1177/2325958219849042 PMC6748475 31109213

[18] Madiba Sphiwe, “When Pregnancy Coincides with Positive Diagnosis of HIV: Accounts of the Process of Acceptance of Self and Motherhood among Women in South Africa,” International Journal of Environmental Research and Public Health 18, no. 24 (December 9, 2021): 13006. 10.3390/ijerph182413006 PMC8700982 34948615

[19] Andrew Lingen-Stallard Christine Furber, and Lavender Tina, “Testing HIV Positive in Pregnancy: A Phenomenological Study of Women s Experiences,” Midwifery 35 (April 1, 2016): 31–38. 27060398 10.1016/j.midw.2016.02.008

[20] Hrdy DB. Cultural practices contributing to the transmission of human immunodeficiency virus in Africa. Reviews of infectious diseases. 1987 Nov 1;9(6):1109–19. 3321361 10.1093/clinids/9.6.1109

[21] Odiachi A, Erekaha S, Cornelius LJ, Isah C, Ramadhani HO, Rapoport L, Sam-Agudu NA. HIV status disclosure to male partners among rural Nigerian women along the prevention of mother-to-child transmission of HIV cascade: a mixed methods study. Reprod Health. 2018 Mar 2;15(1):36. doi: 10.1186/s12978-018-0474-y. 29499704 PMC5833030

[22] Denise Proudfoot RP. Frozen in a Moment in Time: The Experiences of Mothers Being Diagnosed With HIV Infection. 10.1016/j.jana.2017.10.003 29249354

[23] Moshoeshoe M, Madiba S. Parenting the child with HIV in limited resource communities in South Africa: Mothers with HIV’s emotional vulnerability and hope for the future. Women’s Health. 2021 Nov;17:17455065211058565. 10.1177/17455065211058565 PMC8593292 34775847

[24] Angela Kwartemaa Acheampong Florence Naab, and Kwashie Adzo, “Qualitative Exploration of Psychological Reactions and Coping Strategies of Breastfeeding Mothers Living with HIV in the Greater Accra Region of Ghana,” International Breastfeeding Journal 12, no. 1 (June 24, 2017): 28. 10.1186/s13006-017-0119-8 PMC5483253 28652860

[25] Ashaba S, Kaida A, Coleman JN, Burns BF, Dunkley E, O’Neil K, Kastner J, Sanyu N, Akatukwasa C, Bangsberg DR, Matthews LT. Psychosocial challenges facing women living with HIV during the perinatal period in rural Uganda. PloS one. 2017 May 1;12(5):e0176256. 28459866 10.1371/journal.pone.0176256 PMC5411062

[26] Contreras C, Rumaldo N, Lindeborg MM, Mendoza M, Chen DR, Saldaña O, Wong M, Muñoz M, Schrier E, Lecca L, Castro A. Emotional experiences of mothers living with HIV and the quest for emotional recovery: a qualitative study in Lima, Peru. The Journal of the Association of Nurses in AIDS Care: JANAC. 2019 Jul;30(4):440. 31241508 10.1097/JNC.0000000000000051 PMC8324040

[27] Payán DD, Derose KP, Fulcar MA, Farías H, Palar K. “It was as though my spirit left, like they killed me”: the disruptive impact of an HIV-positive diagnosis among women in the Dominican Republic. Journal of the International Association of Providers of AIDS Care (JIAPAC). 2019 May 20;18:2325958219849042. 31109213 10.1177/2325958219849042 PMC6748475

[28] Ashaba Scholastic , “Understanding Coping Strategies during Pregnancy and the Postpartum Period: A Qualitative Study of Women Living with HIV in Rural Uganda,” BMC Pregnancy and Childbirth 17, no. 1 (December 2017): 138. 28482821 10.1186/s12884-017-1321-9 PMC5423027

[29] Madiba S. When pregnancy coincides with positive diagnosis of hiv: Accounts of the process of acceptance of self and motherhood among women in South Africa. International Journal of Environmental Research and Public Health. 2021 Dec 9;18(24):13006. 34948615 10.3390/ijerph182413006 PMC8700982

[30] Iveniuk J, Calzavara L, Bullock S, Mendelsohn JB, Burchell A, Bisaillon L, Daftary A, Lebouché B, Masching R, Thompson T. Social capital and HIV-serodiscordance: Disparities in access to personal and professional resources for HIV-positive and HIV-negative partners. SSM-population health. 2022 Mar 1;17:101056. 35342785 10.1016/j.ssmph.2022.101056 PMC8943292

[31] Natamba Barnabas K. , “The Association between Food Insecurity and Depressive Symptoms Severity among Pregnant Women Differs by Social Support Category: A Cross-Sectional Study,” Maternal & Child Nutrition 13, no. 3 (July 2017). 10.1111/mcn.12351 PMC6865987 27507230

[32] Seffren Victoria , “Association between Coping Strategies, Social Support, and Depression and Anxiety Symptoms among Rural Ugandan Women Living with HIV/AIDS,” AIDS care 30, no. 7 (July 2018): 888–895. 10.1080/09540121.2018.1441969 PMC9850497 29471677

[33] Matsumoto Shoko , “Social Support as a Key Protective Factor against Depression in HIV-Infected Patients: Report from Large HIV Clinics in Hanoi, Vietnam,” Scientific reports 7, no. 1 (2017): 1–12. 10.1038/s41598-017-15768-w PMC5686163 29138432

[34] Xu Jun-Fang , “Family Support, Discrimination, and Quality of Life among ART-Treated HIV-Infected Patients: A Two-Year Study in China,” Infectious Diseases of Poverty 6, no. 1 (November 21, 2017): 152. 10.1186/s40249-017-0364-5 PMC5697335 29157301

[35] Ashaba , “Understanding Coping Strategies during Pregnancy and the Postpartum Period.” 10.1186/s12884-017-1321-9 PMC5423027 28482821

[36] Rogers Anna Joy , “Couple Interdependence Impacts HIV-Related Health Behaviours among Pregnant Couples in Southwestern Kenya: A Qualitative Analysis,” Journal of the International AIDS Society 19, no. 1 (November 24, 2016): 21224. 10.7448/IAS.19.1.21224 PMC5124108 27887669

[37] Berg Rigmor C., Page Samantha, and Øgård-Repål Anita, “The Effectiveness of Peer-Support for People Living with HIV: A Systematic Review and Meta-Analysis,” PLoS ONE 16, no. 6 (June 17, 2021): e0252623. 10.1371/journal.pone.0252623 PMC8211296 34138897

[38] G Visalli , “Knowledge of Sexually Transmitted Infections and Risky Behaviours: A Survey among High School and University Students,” Journal of preventive medicine and hygiene 60, no. 2 (2019): E84. 10.15167/2421-4248/jpmh2019.60.2.1079 PMC6614571 31312737

[39] Mazambara Fine , “Utility of HIV Support Groups in Advancing Implementation Research in Resource-Limited Settings: Experiences from an Urban-Setting HIV Support Group in Zimbabwe,” AIDS Research and Therapy 19, no. 1 (February 14, 2022): 7. 10.1186/s12981-022-00431-w PMC8843025 35164769

